# Twenty-five metagenome-assembled genomes recovered from the gut microbiome of the domestic ferret, *Mustela putorius*

**DOI:** 10.1128/MRA.00364-23

**Published:** 2023-10-19

**Authors:** Kaela K. Amundson, Barbara A. Wolfe, Michael J. Wilkins

**Affiliations:** 1 Department of Soil & Crop Sciences, Colorado State University, Fort Collins, Colorado, USA; 2 Department of Clinical Sciences, Colorado State University, Fort Collins, Colorado, USA; University of Maryland School of Medicine, Baltimore, Maryland, USA

**Keywords:** metagenomics, domestic ferrets, gut microbiome, *Clostridia*, host-microbe interactions

## Abstract

With the advent of metagenomics has come an increased appreciation for the gut microbiome’s role in overall health of mammalian organisms. Even so, studies characterizing taxonomic and functional diversity of the ferret gut microbiome remain limited. Here, we present 25 metagenome-assembled genomes recovered from the gut microbiome of domestic ferrets.

## ANNOUNCEMENT

The domestic ferret, *Mustela putorius*, is a small mammal model organism and close relative of the endangered North American black-footed ferret (*Mustela nigripes*). Ferrets can become infected and transmit many of the same viral diseases as humans and are, therefore, a useful model organism to study the effects of viral infection on host health (1, 2). The gut microbiome is also a key component to overall host health, and therefore, studies investigating microbe-host interactions are of additional benefit. Insights gained by studying the connection between health and the microbiome in domestic ferrets may aid ongoing reintroduction and conservation efforts for the endangered black-footed ferret, which is susceptible to many of the same diseases (3, 4). However, metagenomic studies interrogating the ferret microbiome have been limited to the upper respiratory tract (5), and very few studies using amplicon sequencing or biochemical tests have investigated the gut microbiome (6 – 8). Here, we present 25 metagenome-assembled genomes (MAGs) recovered from the gut microbiome of three domestic ferrets.

Rectal swabs were used to collect samples from the caudal 3 cm of the rectum of the three local domestic ferrets through Colorado State University Veterinary Clinic: MP-FH-1 (female, healthy, 4 mo old), MP-MU-2 (male, unhealthy, 1.5 yr old), and MP-MU-3 (male, unhealthy, 6.3 yr old). DNA was extracted using the Zymobiomics Quick-DNA Fecal/Soil Microbe Kits (Zymo Research, CA, USA). Nucleic acids were sequenced at the Genomics and Microarray Core at the University of Colorado via library preparation with Illumina Nextera XT Library System and metagenomic sequencing via Illumina NovaSeq. Paired-end sequences were trimmed with Sickle (https://github.com/najoshi/sickle) and assembled using MEGAHIT (v1.2.9) (9). Multiple assembly methods were conducted on all samples to maximize genome recovery (Table 1, Zenodo). Only contigs >5,000 bp were used in binning MAGs with MetaBAT2 (10). CheckM (v1.1.3) lineage workflow was used to assess completion and contamination for each MAG (11), with only medium- and high-quality MAGs retained (>50% completion and <10% contamination). The resulting 25 unique MAGs were determined by dRep (12) (v2.2.3) using default parameters. Finally, MAGs were taxonomically classified using GTDB-Tk (13) (v1.0.2), and genes were annotated with DRAM (14) (v1.0.5) with default parameters.

**TABLE 1:** Twenty-five metagenome-assembled genomes recovered from the gut microbiome of black-footed ferrets

MAG ID	MAG GenBank ID	Metagenome NCBI SRA link	Host ferret	Ferret sex	MAG size (bp)	# reads in correspond. metagenome	MAG GC (%)	MAG coverage	MAG complet. (%)	MAG contam. (%)	Taxonomy (genus)
MP-FH-1_10p_bin.1	GCA_029852125.1	SRX17363689	MP-FH-1	F	1653291	134,122,912	37.7	169.2	87.92	0.47	g_Streptococcus
MP-FH-1_10p_bin.2	GCA_029852105.1	SRX17363689	MP-FH-1	F	1841448	134,122,912	38.6	119.9	69.64	0.31	g_Enterococcus_B
MP-FH-1_10p_bin.3	GCA_029852055.1	SRX17363689	MP-FH-1	F	1999268	134,122,912	26.2	184.3	93.85	0	f_CAG-274
MP-FH-1_10p_bin.4	GCA_029852085.1	SRX17363689	MP-FH-1	F	3317916	134,122,912	28	1235	99.19	0	g_Clostridium_P
MP-FH-1_bin.10	GCA_029852045.1	SRX17363689	MP-FH-1	F	1049701	134,122,912	39.2	67.85	59.77	1.55	g_Leuconostoc
MP-FH-1_bin.6	GCA_029852025.1	SRX17363689	MP-FH-1	F	2527940	134,122,912	27.1	63.56	76.88	0	g_Clostridium_P
MP-FH-1_bin.9	GCA_029851965.1	SRX17363689	MP-FH-1	F	3397047	134,122,912	46.1	65.15	78.75	3.96	g_Pedobacter
MP-MU-2_bin.16	GCA_029851955.1	SRX17363690	MP-MU-2	M	1926258	343,529,138	40.7	14.74	91.07	0.45	g_Rodentibacter
MP-MU-2_bin.40	GCA_029852005.1	SRX17363690	MP-MU-2	M	1653420	343,529,138	38.4	11.78	86.78	0	g_Veillonella_A
MP-MU-2_bin.44	GCA_029851945.1	SRX17363690	MP-MU-2	M	2651322	343,529,138	27.1	20.57	50	0	g_Terrisporobacter
MP-MU-2_bin.55	GCA_029851925.1	SRX17363690	MP-MU-2	M	1259317	343,529,138	43.2	20.10	52.59	0	g_Streptococcus
MP-MU-2_bin.67	GCA_029851865.1	SRX17363690	MP-MU-2	M	3329957	343,529,138	28.8	36.38	55.34	7.76	g_Clostridium
MP-MU-2_bin.69	GCA_029851875.1	SRX17363690	MP-MU-2	M	2448635	343,529,138	69.6	27.65	77.19	1.75	g_Actinomyces
MP-MU-2_bin.93	GCA_029851835.1	SRX17363690	MP-MU-2	M	2191856	343,529,138	32.1	24.05	75.61	0	g_Clostridium_J
MP-MU-2_sub_bin.2	GCA_029851885.1	SRX17363690	MP-MU-2	M	2625649	343,529,138	47.7	201.8	95.53	0.48	g_Roseburia
MP-MU-2_sub_bin.3	GCA_029851825.1	SRX17363690	MP-MU-2	M	2568186	343,529,138	34.9	93.49	68	3.86	g_Niameybacter
MP-MU-2_sub_bin.5	GCA_029851795.1	SRX17363690	MP-MU-2	M	1536271	343,529,138	26.2	110.1	81.94	0	f_CAG-274
MP-MU-3_10p_bin.3	GCA_029851825.1	SRX17363691	MP-MU-3	M	1038943	355,510,006	34.3	356.7	60.34	0	g_Lactobacillus
MP-MU-3_10p_bin.6	GCA_029851735.1	SRX17363691	MP-MU-3	M	2924711	355,510,006	28.1	169.7	81.03	1.72	g_Clostridium_P
MP-MU-3_10p_bin.9	GCA_029851745.1	SRX17363691	MP-MU-3	M	1467516	355,510,006	37.1	347.1	73.7	0	g_Lactobacillus
MP-MU-3_bin.25	GCA_029851695.1	SRX17363691	MP-MU-3	M	1292987	355,510,006	40.7	46.14	91.23	0.07	g_Limosilacto bacillus
MP-MU-3_bin.29	GCA_029851665.1	SRX17363691	MP-MU-3	M	3354407	355,510,006	27.9	17.31	98.39	6.45	g_Clostridium
MP-MU-3_bin.4	GCA_029851725.1	SRX17363691	MP-MU-3	M	2578755	355,510,006	37.7	227.1	99.63	0	g_Enterococcus
MP-MU-3_bin.58	GCA_029851645.1	SRX17363691	MP-MU-3	M	2161410	355,510,006	34	10.59	64.91	1.75	g_Cellulosilyticum
MP-MU-3_bin.9	GCA_029851655.1	SRX17363691	MP-MU-3	M	1054999	355,510,006	32.1	48.39	84.82	0	g_Ligilactobacillus

The representative MAGs recovered were primarily affiliated with the Class *Clostridia* (*n* = 12) and the Order *Lactobacillales* (*n* = 9) (Table 1). Other phyla represented were *Actinobacteriota*, *Bacteroidota*, *Firmicutes C*, and *Proteobacteria*. Many of these phyla have also been reported in other studies of the ferret gut microbiome and likely play important roles in the gut microbiome that may influence overall host health (7, 8). Notably, we recovered two MAGs affiliated with *Clostridium perfringens*, a species which is associated with gastroenteritis in endangered black-footed ferrets and presents a considerable challenge to conservation efforts (15). Together, the genomes obtained by this study broaden the taxonomic and metabolic characterization of the ferret gut microbiome.

## Data Availability

Raw metagenomic reads and 25 MAGs have been deposited at NCBI, under BioProject PRJNA875084. Additional metadata can be found on Zenodo 10.5281/zenodo.7613551.
